# Trend and causes of adult mortality in Kersa health and demographic surveillance system (Kersa HDSS), eastern Ethiopia: verbal autopsy method

**DOI:** 10.1186/s12963-017-0144-2

**Published:** 2017-07-01

**Authors:** Wondimye Ashenafi, Frehywot Eshetu, Nega Assefa, Lemessa Oljira, Melkamu Dedefo, Desalew Zelalem, Negga Baraki, Melake Demena

**Affiliations:** 1 Department of Public Health, Haramaya University, Harar, Ethiopia; 2 Department of Nursing, Haramaya University, Harar, Ethiopia; 3 Department of Environmental Health, Haramaya University, Harar, Ethiopia; 4 Department of Statistics, Haramaya University, Harar, Ethiopia; 5 Kersa Health and Demographic Surveillance System (Kersa- HDSS), Haramaya University, Harar, Ethiopia; 6 Center for Disease Control and Prevention, CDC-Ethiopia, Addis Ababa, Ethiopia; 7 School of Mathematics, Statistics and Computer Science, University of KwaZulu-Natal, Durban, South Africa

**Keywords:** Adult mortality, Verbal autopsy method, Adult cause of death, Kersa HDSS, Mortality surveillance

## Abstract

**Background:**

The health problems of adults have been neglected in many developing countries, yet many studies in these countries show high rates of premature mortality in adults. Measuring adult mortality and its cause through verbal autopsy (VA) methods is becoming an important process for mortality estimates and is a good indicator of the overall mortality rates in resource-limited settings. The objective of this analysis is to describe the levels, distribution, and trends of adult mortality over time (2008-2013) and causes of adult deaths using VA in Kersa Health and Demographic Surveillance System (Kersa HDSS).

**Methods:**

Kersa HDSS is a demographic and health surveillance and research center established in 2007 in the eastern part of Ethiopia. This is a community-based longitudinal study where VA methods were used to assign probable cause of death. Two or three physicians independently assigned cause of death based on the completed VA forms in accordance with the World Health Organization’s International Classification of Diseases. In this analysis, the VA data considered were of all deaths of adults age 15 years and above, over a period of six years (2008–2013). The mortality fractions were determined and the causes of death analyzed. Analysis was done using STATA and graphs were designed using Microsoft Excel.

**Results:**

A total of 1535 adult deaths occurred in the surveillance site during the study period and VA was completed for all these deaths. In general, the adult mortality rate over the six-year period was 8.5 per 1000 adult population, higher for males (9.6) and rural residents (8.6) than females (7.5) and urban residents (8.2). There is a general decrease in the mortality rates over the study period from 9.4 in 2008–2009 to 8.1 in 2012–2013. Out of the total deaths, about one-third (32.4%) occurred due to infectious and parasitic causes, and the second leading cause of death was diseases of circulatory system (11.4%), followed by gastrointestinal disorders (9.2%). Tuberculosis (TB) showed an increasing trend over the years and has been the leading cause of death in 2012 and 2013 for all adult age categories (15–49, 50–64, and 65 years and over). Chronic liver disease (CLD) was indicated as leading cause of death among adults in the age group 15–49 years.

**Conclusion:**

The increasing TB-related mortality in the study years as well as the relative high mortality due to CLD among adults of age 15–49 years should be further investigated and triangulated with health service data to understand the root cause of death.

## Background

National and international policymakers, public health officials, and medical personnel need information about the global distribution of deaths by cause in order to set research goals, budgetary priorities, and policies, as well as for planning health care needs. Yet, only 34 countries - representing 15% of the world population - have high-quality cause of death data and almost all of these data are derived from high-income countries. Eighty-five countries representing 65% of the world’s population produce lower quality cause of death data and 74 countries have no such data at all [1]. The lack of such cause-specific mortality data exists mostly in countries with the highest burden of disease. This is largely due to the absence of a well-functioning vital registration system in which every death in the population is medically certified and linked to its causal antecedent [2].

In the absence of such well-functioning vital registration systems, continuous mortality surveillance in a nationally representative sample of the population using a verbal autopsy (VA) method is useful for monitoring mortality trends over time and differentials between subgroups [3]. Verbal autopsy is an indirect method of determining cause of death based on information pertaining to symptoms and signs obtained from a relative or care giver who closely attended to the deceased person prior to his/her death [4].

The health problems of adults have been neglected in many developing countries and a relatively small proportion of public health research in developing societies has been devoted to characterizing the normal course of life through middle age. This is related to the fact that childhood mortality is high and the common assumption is that once childhood is survived, the survival disadvantage is lowered [5]. However, studies in developing countries particularly in sub-Saharan Africa have shown high rates of premature mortality in adults [6, 7]. According to the UN report on the world population in 2014, the probability of dying between ages 15 and 60 was highest in Africa, at 296 per 1000 15-year-olds and the lowest in North America, at 99 per 1000 [8].

In Ethiopia, few mortality statistics showed high proportion of adult deaths. For instance, the 2007 national census indicated an adult mortality incidence of 7.3 per 1000 population at the country level [9]. Evidence from two Ethiopian HDSS mortality studies also revealed a high rate and an increasing trend of adult mortality with greater proportion of burden of deaths attributed to communicable causes [10, 11]. This is similar to the average adult death rate statistics from sub-Saharan Africa where a rise in adult mortality of 4% per year has been noted since HIV become prevalent in the region [6, 12].

The objective of this analysis is to investigate levels, distribution, and trends of mortality over time (2008–2013) as well as the causes of death among adults aged 15 years and older using VA methods in a predominantly rural Kersa HDSS community, eastern Ethiopia.

## Methods

### Study settings and period

The surveillance was undertaken in Kersa HDSS, a member of INDEPTH Network, a worldwide network of HDSSs in Africa, South America, and Asia. Kersa is a district in the eastern part of Ethiopia. According to the Ethiopian government census in 2007, the district has a total population of 172,626, out of which 6.87% are urban dwellers [9].

Kersa HDSS was set up in September 2007 with a baseline census to register all individuals and households in the study area. After the first census, a continuous registration system (CRS) for demographic and health related events have been operational in the whole of the HDSS area. The HDSS has a population of about 60,694 individuals based on 2013 mid-year population estimates [13]. This study uses a longitudinal population based surveillance design and reports mortality trends and adult causes of death using verbal autopsy for the study site for six consecutive years of surveillance, from 2008 to 2013.

### Fieldwork procedure

Age-specific VA questionnaires (neonates, children between four weeks and 14 years of age, and 15 years and above) modified and adapted from Verbal Autopsy Interviewer’s Manual, Sample Vital Registration with Verbal Autopsy (SAVVY), MEASURE Evaluation Project, and US Census Bureau were used to collect information on causes of death. The questionnaire was translated into local languages, Oromiffa and Amharic, and translated back into English to check for consistency**.**

In Kersa HDSS, the verbal autopsies are done by data collectors separately from the regular surveillance data collection. The regular surveillance data collectors are named as vital event enumerators whereas the VA data is collected by verbal autopsy interviewers. The two field workers work in an inter-related manner in death registration and VA data collection. Generally, the process of cause of death assignment begins with a report of a death occurring to a resident of the area recorded by the vital event interviewers through the death recording and reporting form. The death information will be transferred to the VA interviewers who will then complete the VA interview. The vital event interviewers also help the VA interviewers in arranging interview appointment with the deceased family, taking into consideration 45 days for the mourning period [13].

Four individuals who have completed at least a high school education and who have been working in the field as vital event interviewers during the last year have continued to work as VA interviewers. Essentially, they are residents of the surveillance villages who can speak the local languages (Oromiffa and Amharic languages) and have received three days of training every year on the VA questionnaires, which includes recording, contacting close relatives, and data collection procedures. Seven research team members from Haramaya University coordinate the field activities/operations of all vital events registration and VA interviews and are responsible in providing trainings and supervisory support to events, data collectors, and VA interviewers as well as for field supervisors [13].

Two physicians independently reviewed the completed VA questionnaires and assigned codes and titles, with up to three causes of death (underlying, immediate, and contributing factor) using the WHO International Classification of Disease (ICD-10) code and title and then converted into VA code and title. The agreements between the two physician diagnoses on underlying cause were checked by the members of the surveillance team. When there were disagreements in diagnosis, a third physician was assigned to review the cases and final diagnosis was assigned based on the agreement between any of the two physicians. However, if the three physicians assigned different diagnosis, the case was labeled as undetermined or undecided. The three physicians received a three-day training on VA diagnosis and coding procedures in accordance with ICD-10 VA coding system, using ICD-10 training and instructional manual [14]. In this report, for classification of causes of death, the ICD-10 classification in the WHO global burden of disease estimates from 2000 to 2011 was used [15].

As part of data quality assurance, the field supervisors selected 5% of the completed questionnaires and revisited the houses where the VA data were collected to check whether the information was accurate. In the process, any error noted including inconsistent or incomplete data were communicated immediately to the VA interviewers, or in cases where the errors needed due attention, were brought up for discussion during the regular weekly field staff meetings. The supervisors verified that VA data were also checked by the physician VA reviewers, while also assigning cause of death before sending for data entry. Once the VA data passed these steps and were entered into the database, the hard copies were archived in the Kersa HDSS’s data management unit.

### Data analysis

We analyzed the mortality data from the complete census of adult deaths in the study period using STATA and designed graphs using Microsoft Excel.

Deaths were tabulated by socio-demographic characteristics (age at death, year at death, sex, education, marital status, occupation, place of death, and place of residence) and mortality fractions were calculated for each characteristic.

For analysis of broad-cause mortality, all deaths in the population including unspecified and undetermined causes were included in the denominator for the calculation of mortality fractions. For analysis of cause-specific mortality, individuals who died because of unspecified and undetermined causes were not included in the denominator for the calculation of mortality fractions.

A two-year cluster comparison of rates, with 2008–2009 as the base year, was used to measure trends in mortality with respect to age, sex, and place of residence of the deceased. For causes of adult deaths, the broad causes of death were first determined and further sub-divided into specific causes of deaths. Moreover, both broad- and cause-specific mortality fractions were presented for three categories of adult ages (young adults 15–49 years, middle-aged adults 50–64 years, and older adults of age 65 or more years) in accordance with the WHO VA adult age groupings [16].

## Results and discussion

### Results

#### Socio-demographic characteristics

A total of 1535 deaths of adults 15 years of age and above occurred in Kersa health and demographic surveillance site in the six-year period of surveillance (2008–2013). Verbal autopsy was done for all 1535 deaths, and physician review for cause of death assignment was completed for all. About 55% of the deceased were males and a larger proportion (86%) of the deaths were from rural sub-districts (kebeles), and about 82% were not literate. A little more than a third of the deaths occurred among older adults of ages 65 years or more, and about a tenth of the deceased were adults in their youthful ages (15–24 years). About one-third of the deceased (31%) were widowed and 55% were married individuals. The majority (82%) of the deaths occurred at home and only about 11% of the deaths happened at health institutions (Table 1).

**Table 1:** Socio-demographic characteristics of the deceased, Kersa HDSS (2008–2013)

Variable	Category	N	%
Residence	Urban	218	14.2
	Rural	1317	85.8
	Total	1535	100.0
Sex	Male	855	55.7
	Female	680	44.3
	Total	1535	100.0
Age at death in years	15–24	144	9.4
	25–34	193	12.6
	35–44	195	12.7
	45–54	201	13.1
	55–64	259	16.9
	65+	543	35.4
	Total	1535	100.0
Education	Illiterate	1205	82.2
	Elementary	191	13.0
	Secondary and above	70	4.8
	Total	1466	100.0
Occupation	Farmer	655	42.7
	Student	52	3.4
	Housewife	285	18.6
	Other	541	35.3
	Total	1533	100.0
Marital status	Single	151	9.9
	Married	845	55.4
	Widowed	475	31.2
	Divorced	53	3.5
	Total	1524	100.0
Place of death	Health institution	174	11.3
	Home	1260	82.1
	Other place	101	6.6
	Total	1535	100.0

#### Adult mortality rates in Kersa HDSS (2008–2013)

The annual mortality rate for adults 15 years and older in the six years (2008–2013) was 8.5 per 1000 population, higher for males (9.6 per 1000 male population), than females (7.5 per 1000 female population).

The mortality rate was computed by major population characteristics, i.e., age and sex, by three clustered surveillance periods. Comparison by sex and age group generally showed that the proportion dying was higher among men aged 65 years and older throughout the surveillance periods (Table 2).

**Table 2:** Adult mortality rates by age and sex, Kersa HDSS (2008–2013)

		Mortality incidence/1000 population		
		Sex		
Age	Year	Male	Female	Both
	2008–2009	5.4	3.7	4.5
15–49 yrs	2010–2011	4.2	3.2	3.7
	2012–2013	4.6	2.6	3.6
Total	2008–2013	4.7	3.1	3.9
	2008–2009	21.1	16.2	18.7
50–64 yrs	2010–2011	18.2	16.7	17.4
	2012–2013	24.3	16.6	20.5
Total	2008–2013	21.2	16.5	18.9
	2008–2009	98.9	84.6	91.3
65 + yrs	2010–2011	84.5	99.6	92.4
	2012–2013	75.3	55.0	65.3
Total	2008–2013	84.5	76.8	80.6
	2008–2009	10.6	8.3	9.4
All ages	2010–2011	8.3	7.9	8.1
(15+)	2012–2013	9.8	6.3	8.1
Total	2008–2013	9.6	7.5	8.5

During the three-clustered follow-up periods, the overall mortality rate for all ages fell from 9.4 per 1000 adult population in the beginning period of surveillance (2008–2009) to 8.1 per 1000 adult population at the end period (2012–2013), a decline of about 14%. The decline trend was for both men and women, and a sharp decline was observed among individuals aged 65 years or older (from 91.3 in 2008–2009 to 65.3 per 1000 population in 2012–2013) (Table 2).

Overall, an increase in mortality rate was observed with increasing adult age from 3.9 per 1000 population for young adults (15–49 years), to 18.9 rates per 1000 population for middle-aged adults (50–64 years), to 80.6 per 1000 population for older adults (65 or more years) in the surveillance period. Throughout the study years, males had higher death rates than females.

**Fig. 1:**
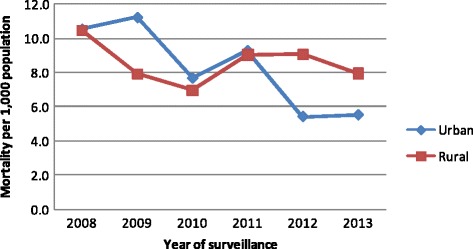
Adult mortality rates (per 1000 population) by place of residence, Kersa HDSS 2008–2013

The mortality rate for urban and rural places of residences for the six surveillance years (2008–2013) were 8.2 per 1000 population and 8.6 per 1000 population, respectively. As can be seen in Fig. 1, both rural and urban mortality rates coincide at the start of the surveillance period at 10.5 per 1000 population. Subsequently, the urban mortality rate became higher for earlier study period (2008 to 2010) and started to take a sharp decline since 2011, and continuing to be lower than the rural mortality rate until the end of the study period (Fig. 1 **).**

#### Causes of death

##### Broad causes of death

Verbal autopsy was done for all 1535 deaths and physician review for cause of death assignment was completed for all. A specific cause of death was assigned for 1107 (72%) of the deaths. The cause of death for 278 (18.1%) of the deaths was unspecified, since the physicians assigning cause of death could not give a specific, probable cause of death based on the information given on the VA questionnaire. 150 (9.8%) of the deaths were labeled undetermined cause of death as there was discordance among the three physician reviewers.

Out of the total deaths, about one-third (32.4%) occurred due to infectious and parasitic causes, diseases of circulatory system was the second leading causes of death (11.4%), and was followed by gastrointestinal disorders (9.2%). (Table 3).

**Table 3:** Broad causes of adult deaths by year, Kersa HDSS, 2008–2013

Year of death				
Broad cause of death	2008–2009	2010–2011	2012–2013	Total
	N (%)	N (%)	N (%)	N (%)
Infectious and parasitic	170 (33.1%)	171 (35%)	156 (29.3%)	497 (32.4%)
Unspecified causes	95 (18.5%)	79 (16.2%)	104 (19.5%)	278 (18.1%)
Diseases of the circulatory	53 (10.3%)	57 (11.7%)	65 (12.2%)	175 (11.4%)
Undetermined	54 (10.5%)	45 (9.2%)	51 (9.6%)	150 (9.8%)
Gastrointestinal disorder	48 (9.4%)	43 (8.8%)	50 (9.4%)	141(9.2%)
External causes of death	23 (4.5%)	31 (6.3%)	37 (6.9%)	91 (5.9%)
Renal disorders	21 (4.1%)	15 (3.1%)	27 (5.1%)	63 (4.1%)
Neoplasm	12 (2.3%)	17 (3.5%)	10 (1.9%)	39 (2.5%)
Pregnancy and childbirth	13 (2.5%)	14 (2.9%)	9 (1.7%)	36 (2.3%)
Respiratory disorders	10 (1.9%)	8 (1.6%)	15 (2.8%)	33 (2.1%)
Nutritional and endocrine	10 (1.9%)	6 (1.2%)	7 (1.3%)	23 (1.5%)
Mental and nervous system	4 (0.8%)	3 (0.6%)	2 (0.4%)	9 (0.6%)
Total	513 (100%)	489 (100%)	533 (100%)	1535 (100%)

**Fig. 2:**
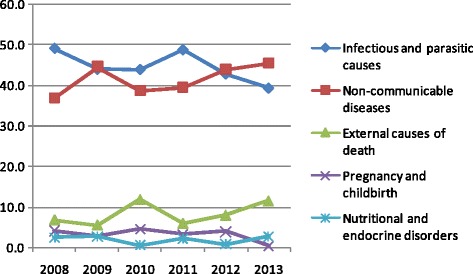
Trend of broad causes of adult death by year, Kersa HDSS, 2008–2013

Re-categorization of the causes of death were split into chronic illness (diseases of circulatory system, gastrointestinal disorders, mental and nervous system disorders, neoplasm, renal disorders, and respiratory disorders), infectious and parasitic causes, external causes, nutritional and endocrine disorders, and pregnancy and childbirth-related deaths. The cause of death fractions with respect to these cause of death groupings showed that infectious and parasitic causes and chronic illnesses are the first and second leading causes of mortality respectively, with a wide range of percentage difference from the other cause of death categories. (Figure 2).

##### Specific causes of death

From the total 1107 adult deaths with assigned specific causes of death, 364 (32.9%), 365 (33.0%) and 378 (34.2%) were observed in the years 2008–2009, 2010–2011, and 2012–2013, respectively. During the surveillance period from 2008 to 2013, intestinal infectious disease including diarrhea was the top leading cause of death (16.5%) followed by tuberculosis (11.6%). Death due to tuberculosis showed a consistent increase in the years and it was the leading cause of death in 2012 and 2013. Chronic liver disease is the third leading cause of death (8.8%) (Table 4).

**Table 4:** Top 20 specific causes of death by year of death, from a total of deceased adults with assigned cause of death (*N* = 1107), Kersa HDSS, Ethiopia, 2008–2013

	Year of death			
Specific cause of death	2008–2009	2010–2011	2012–2013	Total
	N (%)	N (%)	N (%)	N (%)
Intestinal infectious disease (including diarrhea)	61 (16.8%)	76 (20.1%)	46 (12.2%)	183 (16.5%)
Tuberculosis	26 (7.1%)	43 (11.8%)	59 (15.6%)	128 (11.6%)
Chronic liver disease	30 (8.2%)	31 (8.5%)	36 (9.5%)	97 (8.8%)
Acute lower respiratory infection including pneumonia	36 (9.9%)	24 (6.6%)	23 (6.1%)	83 (7.5%)
Congestive heart failure	22 (6.0%)	22 (6.0%)	25 (6.6%)	69 (6.2%)
Cerebrovascular disease	23 (6.3%)	18 (4.9%)	26 (6.9%)	67 (6.1%)
Renal failure	19 (5.2%)	15 (4.1%)	25 (6.6%)	59 (5.3%)
Ischemic heart disease	8 (2.2%)	15 (4.1%)	9 (2.4%)	32 (2.9%)
Malaria	10 (2.8%)	6 (1.6%)	14 (3.7%)	30 (2.7%)
Asthma	9 (2.5%)	7 (1.9%)	13 (3.4%)	29 (2.6%)
Viral hepatitis	23 (6.3%)	2 (0.6%)	1 (0.3%)	26 (2.4%)
Gastric and duodenal ulcer	11 (3.0%)	6 (1.6%)	8 (2.1%)	25 (2.3%)
Assault	2 (0.6%)	8 (2.2%)	10 (2.7%)	20 (1.8%)
Other transport accident	4 (1.1%)	5 (1.4%)	11 (2.9%)	20 (1.8%)
Meningitis	4 (1.1%)	8 (2.2%)	7 (1.9%)	19 (1.7%)
Postpartum hemorrhage	7 (1.9%)	6 (1.6%)	5 (1.3%)	18 (1.6%)
HIV/AIDS	7 (1.9%)	7 (1.9%)	2 (0.5%)	16 (1.5%)
Diabetes mellitus	7 (1.9%)	3 (0.8%)	4 (1.1%)	14 (1.3%)
Malignant neoplasm of esophagus	5 (1.4%)	6 (1.6%)	2 (0.5%)	13 (1.2%)
Paralytic ileus and intestinal obstruction	4 (1.1%)	4 (1.1%)	5 (1.3%)	13 (1.2%)

**Fig. 3:**
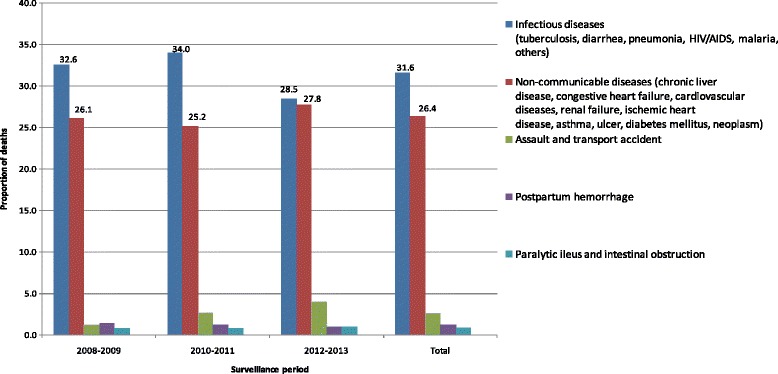
Trends in grouped causes of death by surveillance period, Kersa HDSS, 2008–2013

Re-categorization of the specific causes of death to infectious, non-communicable, and other causes shown in Fig. 3, indicates that infectious diseases such as diarrheal diseases, tuberculosis, malaria, HIV/AIDS, pneumonia, and other infectious illnesses are the leading causes of death, accounting for a total of 31.6% of the deaths. This is followed by non-communicable causes such as chronic liver disease (CLD), congestive heart failure (CHF), cardiovascular diseases (CVD), renal failure (RF), ischemic heart diseases (IHD), asthma, diabetes mellitus (DM), malignant neoplasms, and gastric or duodenal ulcers which altogether accounts 26.4% of all deaths. The above two disease categories are by far the largest proportion of cause of death out of all other causes. Accidents contributed to 2.6% of the adult deaths, and postpartum hemorrhage caused 1.2% of the total deaths (Fig. 3 **).**

Based on the WHO VA adult age groupings of 15–49, 50–64, and 65 years and over, the highest proportion of death was observed for adults of ages 15 to 49 years (39.6%), followed by those with ages 65 years and older (33.3%), and then those with ages between 15 and 64 years (27.1%). Analysis of the 10 top specific causes of death based on these adult age groupings showed that among the age group 15–49 years, chronic liver disease was the leading cause of mortality (13.7%), followed by tuberculosis (11.4%), and then intestinal infectious diseases (9.1%). Among middle-aged adults (50–64 years of age), intestinal infectious disease was the leading cause of death (17.0%), tuberculosis was the second leading (14.3%), followed by cardiovascular diseases (8%). Among older-aged adults 65 years and above, intestinal infectious diseases were the leading cause of death (24.9%), acute lower respiratory tract infections were the second major cause of death (11.4%) and cerebrovascular diseases were the third common cause of mortality (10.8%). (Table 5).

**Table 5:** Top 10 causes of death for three adult age categories by year, Kersa HDSS, 2008–2013

Age group	Causes of death	Year of death			
		2008–2009	2010–2011	2012–2013	Total
		N (%)	N (%)	N (%)	N (%)
15–49 years	Chronic liver diseases	20 (13.6%)	22 (15.2%)	18 (12.3%)	60 (13.7%)
	Tuberculosis	12 (8.2%)	18 (12.4%)	20 (13.7%)	50 (11.4%)
	Intestinal infectious diseases	11 (7.5%)	13 (9.0%)	16 (11.0%)	40 (9.1%)
	Congestive heart failure	12 (8.2%)	8 (5.5%)	6 (4.1%)	26 (5.9%)
	Assault	2 (1.4%)	8 (5.5%)	9 (6.2%)	19 (4.3%)
	Acute lower respiratory infection	6 (4.1%)	7 (4.8%)	5 (3.4%)	18 (4.1%)
	Other transport accidents	4 (2.7%)	4 (2.8%)	10 (6.8%)	18 (4.1%)
	Postpartum hemorrhage	7 (4.8%)	5 (3.4%)	5 (3.4%)	17 (3.9%)
	Malaria	5 (3.4%)	4 (2.8%)	7 (4.8%)	16 (3.7%)
	Gastric and duodenal ulcer	6 (4.1%)	4 (2.8%)	4 (2.7%)	14 (3.2%)
	Total	85 (57.8%)	93 (64.1%)	100 (68.5%)	278 (63.5%)
50–64 years	Intestinal infectious diseases	15 (16.1%)	22 (22.4%)	14 (12.8%)	51 (17.0%)
	Tuberculosis	8 (8.6%)	15 (15.3)	20 (18.3%)	43 (14.3%)
	Cerebrovascular diseases	8 (8.6%)	9 (9.2%)	7 (6.4%)	24 (8.0%)
	Acute lower respiratory infection	10 (10.8%)	7 (7.1%)	6 (5.5%)	23 (7.7%)
	Chronic liver disease	7 (7.5%)	4 (4.1%)	10 (9.2%)	21 (7.0%)
	Renal failure	2 (2.2%)	6 (6.1%)	11 (10.1%)	19 (6.3%)
	Congestive heart failure	2 (2.2%)	6 (6.1%)	10 (9.2%)	18 (6.0%)
	Ischemic heart disease	3 (3.2%)	7 (7.1%)	4 (3.7%)	14 (4.7%)
	Asthma	1 (1.1%)	5 (5.1%)	6 (5.5%)	12 (4.0%)
	Diabetes mellitus	6 (6.5%)	2 (2.0%)	2 (1.8%)	10 (3.3%)
	Total	62 (66.7%)	83 (84.7%)	90 (82.6%)	235 (78.3%)
≥65 years	Intestinal infectious diseases	35 (28.2%)	41 (33.6%)	16 (13.0%)	92 (24.9%)
	Acute lower respiratory infection	20 (16.1%)	10 (8.2%)	12 (9.8%)	42 (11.4%)
	Cerebrovascular diseases	13 (10.5%)	9 (7.4%)	18 (14.6%)	40 (10.8%)
	Tuberculosis	6 (4.8%)	10 (8.2%)	19 (15.4%)	35 (9.5%)
	Renal failure	11 (8.9%)	6 (4.9%)	10 (8.1%)	27 (7.3%)
	Congestive heart failure	8 (6.5%)	8 (6.6%)	9 (7.3%)	25 (6.8%)
	Chronic liver disease	3 (2.4%)	5 (4.1%)	8 (6.5%)	16 (4.3%)
	Ischemic heart diseases	5 (4.0%)	7 (5.7%)	4 (3.3%)	16 (4.3%)
	Asthma	4 (3.2%)	2 (1.6%)	6 (4.9%)	12 (3.3%)
	Malaria	4 (3.2%)	2 (1.6%)	5 (4.1%)	11 (3.0%)
	Total	109 (87.9%)	100 (81.9%)	107 (86.9%)	316 (85.6%)

### Discussion

This study showed that the overall mortality rate for adults 15 years and older for the surveillance period 2008–2013 was 8.5 per 1000 population. This indicates a higher adult mortality than the Ethiopian 2007 census result, where the adult mortality rate for Oromia region (the region where Kersa district is located) is 7.1 per 1000 population and the national average of 7.3 adult deaths per 1000 population [9].

Males have a higher mortality incidence (9.6 per 1000 population) than females (7.5 per 1000 population) among Kersa HDSS community in the surveillance years. The latest DHS report of Ethiopia indicates a similar sex differentials in the mortality experience revealing higher level of adult mortality among men (5.0 deaths per 1000 population) than among women (4.1 deaths per 1000) [17].

Compared to the early years of surveillance (2008 to 2010), where an inconsistent pattern of mortality was observed in terms of residence, a sharp decline of adult mortality was observed in urban areas since 2011. This might indicate a recent gap in rural disadvantages in terms of factors leading to adult deaths, mainly infectious and parasitic causes that are the leading cause of death in the study area. These factors might include unavailability or poor accessibility of safe drinking water, poor housing and sanitary conditions, and other household characteristics that can contribute to spread of communicable diseases in rural areas, compared to urban areas. However, the inconsistent pattern of mortality observed over the years with respect to place of residence was not in agreement to the finding from Butajira HDSS of central Ethiopia which showed a consistent pattern of higher adult mortality rate among the rural dwellers compared to the urban during the whole period of 1987–2004 [18]. Nonetheless, in terms of specific causes of death, nearly similar proportion of deaths were reported due to CLD among adults of age 15–49 years in both communities (13.7% for Kersa HDSS and 11.3% for Butajira HDSS) [10]. In contrast, CLD was the least contributing cause of death (3.5%) among adults aged 15–49 years in rural communities of Northern Ethiopia [19]. The possible explanation for this might be availability of khat (an indigenous chewing plant used as a stimulant in some parts of Ethiopia, especially in the eastern part of the country), as khat chewing is a risk factor for CLD [20]. This plant is a cash crop in both communities of Kersa and Butajira, compared to the Kilte Awlalo district communities of northern Ethiopia [21, 22].

Infectious and parasitic causes as dominant causes of death among Kersa HDSS community is in agreement with 2004 WHO estimates of the global burden of disease (GBD) for low-income countries where infectious and parasitic diseases (including malaria) were reported to be first among the top 10 leading causes of death [23]. This is also in agreement with a recent report of the 2010 WHO GBD estimates for sub-Saharan Africa where nearly a fifth of deaths were caused by diarrhea, lower respiratory infections, and other common infectious diseases, particularly among young adults [24].

The analysis in this study has also revealed that NCDs were the second leading cause of death (26.4%) next to infectious and parasitic causes (32.4%). This is comparable with the study findings from northern Ethiopia among the population of the Kilete Awlealo HDSS where infectious and parasitic diseases were leading causes of deaths (35.9%), followed by chronic diseases accounting for 28.6% of total deaths, and diseases of circulatory system and neoplasms being the most common [19]. Our finding differs from the Addis Ababa burial surveillance where 51% of deaths were from NCDs, and 42% to communicable diseases [25]. This difference is most likely due to the difference in the study settings, as the study communities of Addis Ababa surveillance are considerably more urban and NCDs are expected to be more prevalent in urban settings.

In this analysis, out of deaths attributed to infectious causes, intestinal infectious diseases including diarrhea were the top leading cause of death followed by tuberculosis. Death due to tuberculosis showed a consistent increase over the years and it became the leading cause of death in 2012 and 2013. Chronic liver disease is the third leading cause of death. This finding is more or less the same as the findings from the Kilete Awlealo DHSS study, which showed among the communicable diseases, tuberculosis was the leading cause of death (12%), followed by intestinal infectious diseases (6.8%) [19]. Similar magnitude of deaths was also reported in the recent study of Addis Ababa burial surveillance, where tuberculosis deaths were reported as high as 12% of all deaths [25] .

Death due to HIV/AIDS is likely to be underestimated in many reports [6, 12, 26]. This might hold true for Kersa HDSS population, as families are unlikely to directly disclose the deceased sero-status or be unwilling to indirectly expound cause of death relating to HIV/AIDS in the VA interviewing due to fear of stigma. Moreover, HIV-related cause of death is most likely obscured by death due to opportunistic illnesses like tuberculosis, pneumonia and meningitis, and these diseases have taken a considerable proportion of deaths in our study.

Generally, the strength of this study is that it used community based quantitative demographic and health surveillance data for a relatively longer period of time (2008 to 2013). One limitation of this report could be the high proportion of “unclassified” (unspecified and undetermined) adult deaths due to lack of sufficient information in the VA data. To have unbiased mortality estimates, however, we excluded these unclassified causes of death in the calculation of cause-specific mortality fractions.

Due to the nature of VA that the data are collected sometime after the person has died, a recall bias might be introduced in data collection process. Moreover, as these VA data have not been supported with clinical findings, it is believed it would introduce information bias. However, to overcome these biases, strict attention was given during data collection to link information with events in the course of illnesses associated to the death and to collect the VA data during a convenient time for the respondent.

## Conclusions and recommendations

In this analysis, males had a higher mortality rate than females in almost all the study years. Similar to other rural HDSS settings of Ethiopia, infectious diseases are the leading causes of adult mortality in Kersa HDSS and this signals that there is a need to strengthen the preventive and curative health intervention on infectious diseases at country level.

Non-communicable diseases were the second leading causes of mortality in Kersa HDSS, with considerably higher proportion of deaths over the last surveillance period (2012 and 2013). This indicates that it requires due attention at all levels of the health sector in designing strategies to address the emerging NCDs. Particularly, among the age group 15–49 years, chronic liver disease was the leading cause of mortality. Consequently, based on this finding, further studies need to be designed to understand the background factors such as burden of hepatitis viruses.

In this study, death due to tuberculosis showed a consistent increase over the study years and was even the leading cause of infectious death in 2012 and 2013. The increasing TB-related mortality should be further investigated and triangulated with health service data to understand the root cause of death as to whether the deceased started TB treatment, was co-infected with HIV (linked to HIV treatment), if there was multi-drug resistance to TB, or if there was TB drug toxicity and the like.

A considerable number of young adults living in Kersa district are at much greater risk of death from chronic diseases such as CLD. Although the primary healthcare system in rural Ethiopia appears to have been promising in dealing with the problems of sanitation and hygiene and maternal and child health, it is less well-equipped to deliver care and prevention for chronic diseases like CLD. Thus, an urgent consideration as to how to address the emerging challenge of non-communicable conditions in the rural part of the country, particularly CLD, is needed.

## Abbreviations

AIDS: Acquired immune deficiency syndrome
BRHP: Butajira rural health project
CDC: Centers for disease control and prevention
CDs: Communicable diseases
CHF: Congestive heart failure
CLD: Chronic liver disease
CSA: Central Statistical Agency
CVDs: Cardiovascular diseases
DHS: Demographic and health survey
DM: Diabetes mellitus
DSS: Demographic surveillance system
EPHA: Ethiopian Public Health Association
GBD: Global burden of disease
HDSS: Health and demographic surveillance system
HIV: Human immunodeficiency virus
ICD: International classification of diseases
IHD: Ischemic heart disease
NCDs: Non-communicable diseases
RF: Renal failure
TB: Tuberculosis
US: United States
VA: Verbal autopsy

## References

[1] WHO: World Health Statistics 2012. Geneva: World Health Organization (WHO); 2012.

[2] Begg S, Rao C, Lopez A (2005). Design options for sample based mortality surveillance. Int J Epidemiol.

[3] Quigley M (2005). Commentary: verbal autopsies-from small-scale studies to mortality surveillance systems. Int J Epidemiol.

[4] Chandramohan D, Setel P, Quigley M (2001). Effect of misclassification of causes of death in verbal autopsy: can it be adjusted?. Int J Epidemiol.

[5] Jamison DT, Feachem RG, Makgoba MW, Bos ER, Baingana FK, Hofman KJ, Rogo KO (2006). Disease and mortality in sub-Saharan Africa: World Bank Washington, DC.

[6] Timaeus I, Momodou J (2004). Adult mortality in sub-saharan Africa: evidence from demographic and health surveys. Demography.

[7] Streatfield PK, Khan WA, Bhuiya A, Hanifi SM, Alam N, Bagagnan CH, Sie A, Zabré P, Lankoandé B, Rossier C, et al. Adult non-communicable disease mortality in Africa and Asia: evidence from INDEPTH Health and Demographic Surveillance System sites. Global health action. 2014;7(1). doi:10.3402/gha.v7.25365. 10.3402/gha.v7.25365 PMC4220128 25377326

[8] UN (2014). The world population situation in 2014: a concise report. Department of Economic and Social Affairs Population Division.

[9] CSA (2010). Population and housing census country 2007.

[10] Lulu K, Berhane Y (2005). The use of simplified verbal autopsy in identifying causes of adult death in a predominantly rural population in Ethiopia. BMC Public Health.

[11] Weldearegawi B, Melaku YA, Spigt M, Dinant GJ. Applying the InterVA-4 model to determine causes of death in rural Ethiopia. Global health action. 2014;7(1). doi:10.3402/gha.v7.25550. 10.3402/gha.v7.25550 PMC4220136 25377338

[12] Chihana M, Floyd S, Molesworth A, Crampin AC, Kayuni N, Price A, Zaba B, Jahn A, Mvula H, Dube A (2012). Adult mortality and probable cause of death in rural northern Malawi in the era of HIV treatment. Tropical Med Int Health.

[13] Assefa N, Oljira L, Baraki N, Demena M, Zelalem D, Ashenafi W, Dedefo M: Profile of Kersa HDSS: the Kersa Health and Demographic Surveillance System. Int J Epidemiol. 2015: doi: 10.1093/ije/dyv284. 10.1093/ije/dyv284 PMC4795560 26510420

[14] WHO: International Statistical Classification of Diseases and Related Health Problems. Tenth Edition (ICD-10), Version for 2010. Volume 2. Instructional Manual. Geneva: World Health Organization (WHO); 2010.

[15] WHO: WHO methods and data sources for global burden of disease estimates 2000–2011 (Global Health Estimates Technical Paper WHO/HIS/HSI/GHE/2013.4). Geneva: World Health Organization (WHO); 2013.

[16] WHO: Verbal autopsy standards: the 2012 WHO verbal autopsy instrument: release candidate 1. Geneva; World Health Organization (WHO); 2012.

[17] CSA (2012). Ethiopia demographic and health survey 2011.

[18] Fantahun M, Berhane Y, Högberg U, Wall S, Byass P (2008). Young adult and middle age mortality in Butajira demographic surveillance site, Ethiopia: lifestyle, gender and household economy. BMC Public Health.

[19] Weldearegawi B, Ashebir Y, Gebeye E, Gebregziabiher T, Yohannes M, Mussa S, Berhe H, Abebe Z (2013). Emerging chronic non-communicable diseases in rural communities of northern Ethiopia: evidence using population-based verbal autopsy method in Kilite Awlaelo surveillance site. Health Policy Plan.

[20] Al-Motarreb A, Al-Habori M, Broadley KJ (2010). Khat chewing, cardiovascular diseases and other internal medical problems: the current situation and directions for future research. J Ethnopharmacol.

[21] Alem A, Kebede D, Kullgren G (1999). The prevalence and socio-demographic correlates of khat chewing in Butajira, Ethiopia. Acta Psychiatr Scand.

[22] Reda AA, Moges A, Biadgilign S, Wondmagegn BY (2012). Prevalence and determinants of khat (*Catha edulis*) chewing among high school students in eastern Ethiopia: a cross-sectional study. PLoS One.

[23] WHO: The Global Burden of Disease: 2004 Update. Geneva: World Health Organization (WHO); 2008.

[24] Institute for Health Metrics and Evaluation (IHME). The Global Burden of Disease: Generating Evidence, Guiding Policy. Seattle, WA: IHME; 2013.

[25] Misganaw A, Mariam DH, Araya T (2013). Association of socioeconomic and behavioral factors with adult mortality: analysis of data from verbal autopsy in Addis Ababa, Ethiopia. BMC Public Health.

[26] Molla MBP, Berhane Y, Lindtjorn B (2008). Mortality decrease among young adults in southern central Ethiopia. Ethiop J Health Dev.

